# Laser Treatment of Cotton Fabric for Durable Antibacterial Properties of Silver Nanoparticles

**DOI:** 10.3390/ma5071247

**Published:** 2012-07-16

**Authors:** Shirin Nourbakhsh, Ali Ashjaran

**Affiliations:** Textile Department, Shahre Rey Branch, Islamic Azad University, P.O. Box 18155-144, Tehran 001-680-000, Iran; E-Mail: a.ashjaran@gmail.com

**Keywords:** laser ablation, silver nanoparticles, durability, cotton, antibacterial

## Abstract

In the present study, cotton fabric was exposed to laser exposure at different energy levels and then the silver nanoparticles were coated on untreated and laser treated cotton fabrics. Methylene blue dye was used to detect the presence of carboxylic acid groups (-COO^−^) on laser treated cotton and the dye absorption results were determined spectrophotometrically. ICP-OES (Inductively Coupled Plasma Optical Emission Spectroscopy) analysis and antibacterial tests were carried out to investigate the silver ion content and bactericidal properties of silver nanoparticles on cotton fabrics. Infrared spectroscopy (FTIR/ATR) analysis and scanning electron microscopy (SEM) were used to identify chemical changes and to study the morphology of the surface of the fibers. EDAX (Energy Dispersive X-ray Spectroscopy) analysis was calculated for SEM micrographs. The results showed according to the higher uptake of methylene blue dye that the negative charge of the carboxylic acid groups had been created by laser treatment. Although the FTIR spectroscopy results did not show an increase in carboxylic acid groups, the cationic dye absorption increased. The durability of the Ag^+^ ion particles on repeated laundered laser treated cotton was proven by antibacterial and ICP tests, particularly when the laser energy was increased.

## 1. Introduction

The growth of microorganisms on textiles can cause problems, therefore, textile products are often treated with antimicrobial agents to prevent the proliferation of microorganisms [1,2]. Silver metal is one such antibacterial material which has been utilized for many years. A very low concentration of silver ions is sufficient for antibacterial effects. Silver nanoparticles have recently been used as a safe antibacterial agent for human health on different materials such as fibers, polymers and antimicrobial textile products. Durability of antimicrobial finishes on textiles is important for consumer usage. Research continues to find ways of increasing lasting antibacterial effects in the fabric. In this study, silver nanoparticles were applied on untreated and laser treated fabrics, the surface of which had been laser modified to investigate the durability of silver ions [3,4,5,6,7,8,9]. The cotton fiber consists of hydroxyl groups which are affected by chemical interactions. The hydroxyl groups are converted to carboxyl and carbonyl groups in the presence of oxidative agents or oxygen plasma discharge [10,11,12,13,14].

Laser is classified as light amplification by stimulated emission of radiation [15]. The optimal characteristics of laser light have increased its application in many fields including medical, industrial, and the military. The benefit of laser is due to its high-speed, more handling safety and unique process applications. The laser used in the textile industry is of great importance and is used for the decoloration of denim [16,17,18], cutting fabrics in industry [15], and wool shrinkage control as laboratory work [19]. There are several commercial lasers in industry, which are used for industrial applications, including, the laser of Nd:YAG, CO_2_ and Excimer [15]. In previous works, coating of silver nitrate and silver nanoparticles on plasma treated and untreated cotton and cotton/polyester fabrics were investigated [3,20,21,22,23,24,25]. The researchers used silver nitrate and silver nano-particles to illustrate the higher absorption of silver ions on nitrogen plasma treated and cationized cotton fabrics [22,24]. In this study, the CO2 laser was used for surface modification of cotton fabrics and then the cotton fabrics were dyed by methylene blue dye for investigation of the existence of carboxylic acid groups. The Infrared spectroscopy analysis (FTIR/ATR) and scanning electron microscopy (SEM) were utilized to determine the functional groups and surface morphology. Silver nanoparticles were applied on untreated and laser treated cotton fabrics. ICP-OES analysis and anti-bacterial tests were obtained in accordance with the standard procedures and the laundering was repeated to determine the durability of anti-bacterial effects on cotton fabrics.

## 2. Results and Discussion

The color parameters (L*, a*, b*), K/S value (color yield) and ΔE (color difference) of dyed fabrics by methylene blue dye are indicated in Table 1. The methylene blue dye is a cationic dyestuff which reacts with anionic substrates and is used for determination of carboxylic acid groups on oxidized cotton [12]. The L* value indicates the lightness of the dyed fabric and a lower amount of lightness means a darker fabric. The untreated cotton fabric showed an L* value of 70.45, and after laser treatment at a power of 1000 W, the L* value decreased to 69.87 and 69.05. The increase of applied laser energy on cotton fabrics caused the higher amount of methylene blue dye absorption due to the reduction of L* value. The color yield of the dyed fabrics is obtained from the following Kubelka-Munk Equation:

K/S = (1 − R)^2^/2R (1)

where R is the reflectance; the K/S value reveals the color yield of the dyed fabric. When the fabric becomes darker, the color yield increases. The color strength (K/S) for untreated cotton fabric was 0.636 and it increased to 0.782 and 0.832 for laser treated cotton fabric at a power of 100 watts and the fabric became darker. It seems that the laser effect at a power of 100 watts was significant for the absorption of cationic dye; this is due to the presence of anionic groups on the cotton surface which might be carboxylic acid groups. Color difference (ΔE) of untreated and laser treated cotton fabric is calculated by the Equation (2) [26]:

(2) $$\Delta E=\sqrt{\Delta L^{2}+\Delta a^{2}+\Delta b^{2}}$$

**Table 1:** Color parameters of methylene blue dyed untreated and laser treated cotton fabric.

Power (w)	100		75		50	Untreated
Scan speed (cm/s)	1000	2000	1000	2000	1000	
L*	69.05	69.87	70.16	70.11	70.41	70.45
A*	−5.49	−5.53	−2.76	−4.58	−2.13	−2.38
B*	−18.41	−18.18	−12.36	−7.45	−14.75	−16.09
K/S	0.832	0.782	0.731	0.651	0.648	0.636
∆ E	4.12	3.82	3.76	2.6	1.36	–

The color difference of laser treated cotton fabrics was calculated as compared to untreated fabric. The color difference increased to 4.12 for laser treated cotton fabric at a high level of energy. The increase in laser power affected on dye absorption as the results of ΔE indicated.

**Figure 1:**
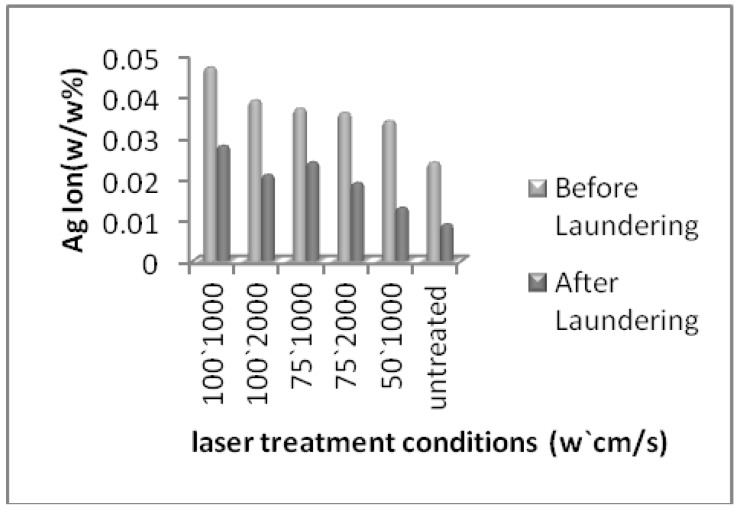
The silver ion content on untreated and laser treated cotton fabrics.

In Figure 1 we can see the Ag ion content on untreated and laser treated cotton fabrics which were obtained by ICP-OES analysis. The untreated cotton fabric showed 0.024% of Ag ion content and it decreased to 0.009% after repeated laundering. The highest amount of observed silver ions was 0.047% for the higher energy level of laser (100 W, 1,000 cm/s) which reduced to 0.028% with repeated laundering. These results show that not only the laser effect and its higher energy level can increase the absorption of silver ions on cotton surface but also the durability of silver ions remained after repeated laundering.

**Figure 2:**
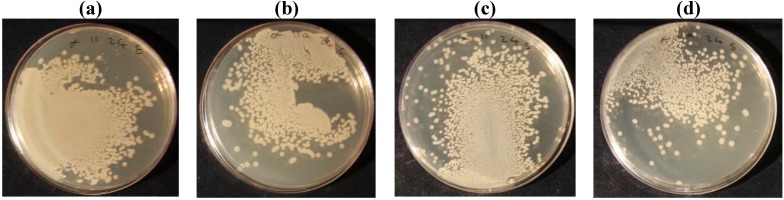
The bacteria colonies of a silver nanoparticles coating on untreated cotton (**a**) Escherichia Coli before laundering; (**b**) Escherichia Coli after laundering; (**c**) Staphylococcus aureus before laundering; (**d**) Staphylococcus aureus after laundering.

**Figure 3:**
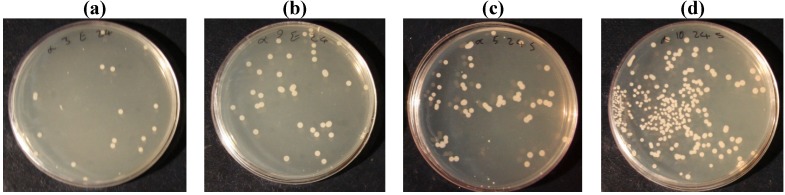
The bacteria colonies of silver nanoparticles coating on laser treated cotton (**a**) Escherichia Coli before laundering; (**b**) Escherichia Coli after laundering; (**c**) Staphylococcus aureus before laundering; (**d**) Staphylococcus aureus after laundering.

Table 2 shows the percentage of bacteria reduction with silver nanoparticles on untreated and laser treated cotton fabrics. The antibacterial tests were performed with two kinds of bacteria; Escherichia- Coli and staphylococcus aureus. Results showed that the silver ion was more effective in reducing Escherichia coli than Staphylococcus aureus bacteria. By increasing the laser energy level, silver ion absorption increased on cotton fabric as the percentage of bacteria reduction reached 99.25% at 100 W and 1,000 cm/s laser conditions and after repeated laundering it decreased to 98.3%. Whereas a silver nanoparticles coating on untreated cotton fabric showed 39.9% of bacteria reduction. Figure 2 and Figure 3 show the colonies of bacteria (E. coli and S. aureus) on untreated and laser treated cotton fabrics at 100 W and 1,000 cm/s. The colonies of bacteria were counted and the percentage of bacteria reduction was calculated using Equation (3).

**Table 2:** Bacteria reduction percentage, Ag content results of EDAX analysis of a silver nanoparticles coating and carboxyl groups’ contents of untreated and laser treated cotton fabrics.

Bacteria	Laser power (w)	100		75		50	Untreated
	Scan speed (cm/s)	1000	2000	1000	2000	1000	
Escherichia Coli	Before laundering	99.25	98.65	98.4	96.45	98.05	39.9
	After laundering	98.3	97.45	96.35	96.05	96.1	26.2
staphylococcus aureus	Before laundering	97.1	91.25	82.5	77.5	69.65	37.5
	After laundering	87	80.25	73.6	68.25	45	23.3
(%) Ag content (EDAX analysis)		5.25	5.03	4.82	4.67	4.61	4.34
Carboxyl groups content (meq/100g)		3.86	3.24	3.12	2.87	2.08	1.36

**Figure 4:**
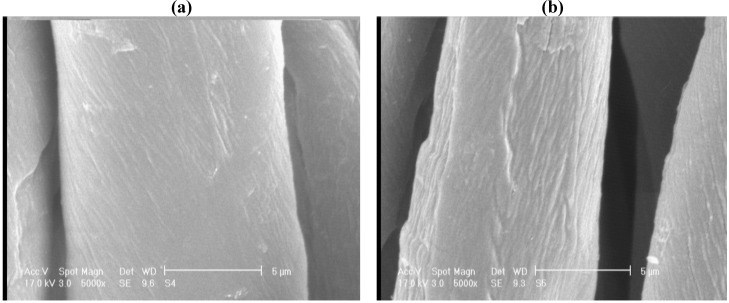

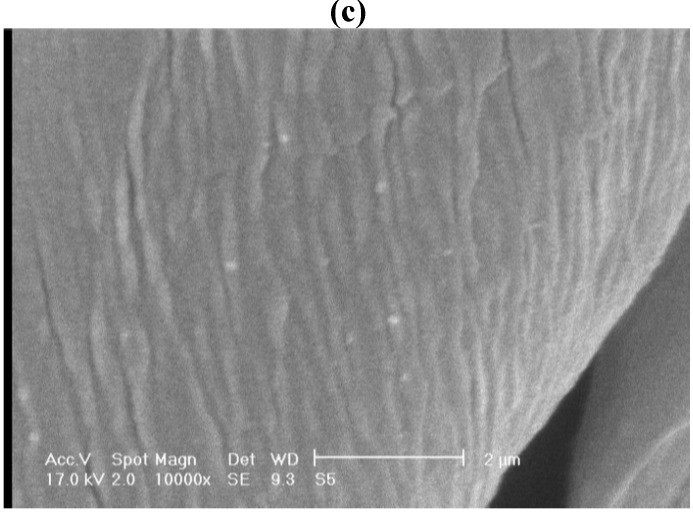
**S**canning electron microscopy (SEM) micrographs of (**a**) untreated cotton; (**b**) laser treated at 100W, 100cm/s, magnification of 5,000; (**c**) laser treated at 100 W, 100cm/s and magnification of 10,000.

**Figure 5:**
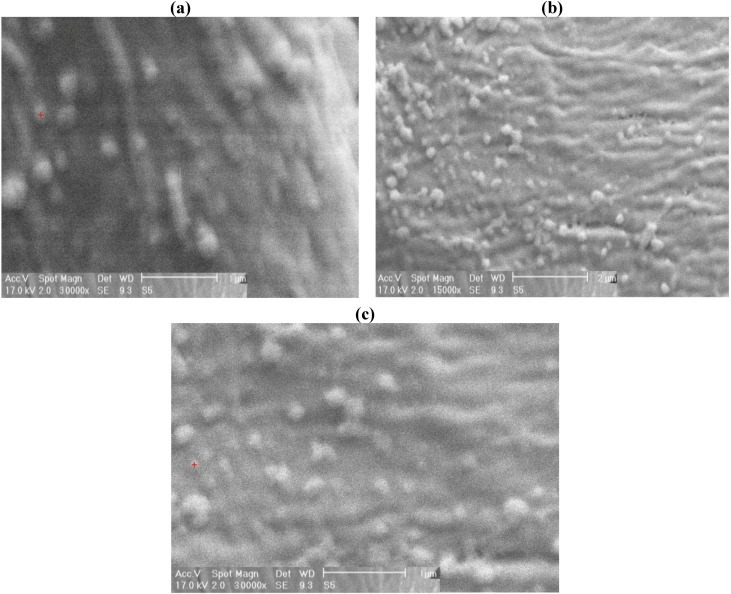
SEM micrographs of **(a)** silver nanoparticles on untreated cotton; **(b)** laser treated at 100W, 100cm/s, magnification of 15,000; **(c)** laser treated at 100 W, 100cm/s and magnification of 30,000.

Figure 4 displays SEM micrographs of untreated and laser treated cotton fabrics at magnifications of 5,000, 10,000 and 15,000. It can be seen that laser treatment of cotton fiber caused some gaps and cracks on the fiber surface. Figure 5 shows the micrographs of cotton fabric treated with silver nanoparticles, the EDAX analysis was obtained from the SEM micrographs (Table 2). The untreated cotton fabric showed 4.34% of silver ion content whereas laser treated cotton fabric showed 5.25% of Ag ions. Energy dispersive X-ray spectroscopy (EDAX) is an analytical technique used for the elemental analysis or chemical characterization of a sample. The EDAX analysis is not an accurate method for determination of silver ion amounts, and it is better to use ICP-OES (Inductively Coupled Plasma Optical Emission Spectroscopy) analytical method for the analysis.

**Figure 6:**
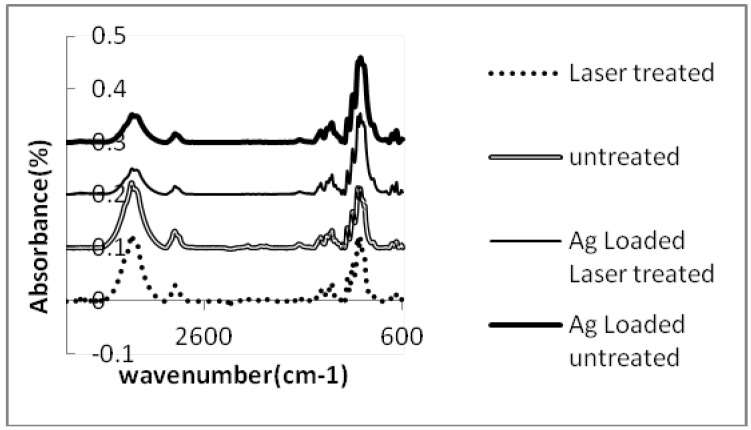
FTIR/ATR spectra of untreated, laser treated and silver nanoparticles coated cotton.

The results of infrared spectroscopy (ATR/FTIR) of the fabrics are illustrated in Figure 6. FTIR/ATR spectroscopy (attenuated total reflectance) indicates the chemical bonding on the fiber surface. The spectra of untreated and laser treated cotton fabrics are similar, although we expected to see the existence of carboxyl acid groups according to the results of the methylene blue dyed fabrics. The spectra of the silver nanoparticles coating on untreated and laser treated cotton fabrics showed a decrease in the intensities of the peaks in the regions of 3,000 cm^−1^ and 3,400 cm^−1^. These peaks are related to the hydroxyl groups of cellulose [27]. The intensities of the peaks are higher for untreated and laser treated cotton fabrics and indicates the hydroxyl groups of cellulose before silver coating whereas after silver nanoparticles loading the intensities of the related peaks reduced. This might be due to a decrease of this group on the fiber surface. Decrease of hydroxyl groups demonstrates physical absorption of silver ions to these groups. The silver ions were deposited on either untreated cotton or laser treated cotton which might present the hydroxyl groups of carboxylic acids on laser treated cotton fabric.

The results of ICP analysis (Figure 1) and anti-bacterial tests (Table 2, Figure 2 and Figure 3) were more significant than the other tests in showing the effect of silver ions on laser treated cotton fabric, so that the ICP results showed the increase in silver ion content with increasing laser power. The ICP results also indicated a higher amount of Ag ion concentration on laser treated cotton fabric as compared to untreated cotton; therefore surface treatment by laser exposure was effective for absorption of Ag^+^ ions. The antibacterial test also showed bacteria reduction on silver loading of laser treated cotton fabrics. Regarding the results of antibacterial tests and ICP-OES analysis, the presence of silver ions on laser treated cotton fabric was certifiable. In FTIR/ATR spectroscopy of the fiber surface (Figure 6) the decrease in the intensities of peaks corresponding to hydroxyl groups is attributed to possible binding of silver ions (Ag^+^). The results of carboxyl group content, obtained by titration of TAPPI standard method, are shown in Table 2. The untreated cotton fabric showed 1.36 meq/100g of carboxylic groups whereas it reached 3.86 for laser treated at 100 W and 1000 cm/s. Laser treatment of cotton fabric could therefore increase the carboxylic acid groups as exemplified by these results.

## 3. Experimental Section

### 3.1. Materials

Plain weave cotton fabric (109 g/m^2^) was used in this study. The used materials were non-ionic detergent Laventin LNB (BASF, Ludwigshafen, Germany), AATCC standard detergent, silver nanoparticles solution (Plasmachem, Berlin, Germany), methylene blue dye (C.I. basic blue 9, Juhua chemical Co. Ltd, Tianjin, China), nitric acid (Merck, Darmstadt, Germany).

### 3.2. Methods

The bleached cotton fabric was prepared for laser surface modification, in that it was washed by non-ionic detergent, then rinsed and dried. Each of the fabrics was cut to 30 × 30 cm^2^ before laser radiation. The used laser was a CO_2_ laser from (LST) laser manufacturing company in Turkey, at powers of 50, 75, and 100 watts and scan speeds of 1,000 and 2,000 cm/s. The laser treated and untreated cotton fabrics were dyed by cationic methylene blue dye. The dyeing was carried out at room temperature for 5 minutes in 50 ml of 1% methylene blue (C.I. basic blue 9, Juhua chemical Co. Ltd, Tianjin, China) dye solution, then rinsed and dried [12]. The color parameters of methylene blue dyed fabrics were investigated with a spectrophotometer (D_65_/10°) to determine the carboxyl groups.

The untreated and laser treated cotton samples, each weighing 1 gram were dipped in 10 cc of nano silver solution at a concentration of 20 ppm for 30 minutes at ambient temperature, then passed through pad rollers, dried at a temperature of 100 °C and cured at a temperature of 150 °C for 1 minute. Then the washing process was carried out in distilled water to remove the non-absorbed fragments of the cotton surface. The water used for the test was distilled water because the tap water contains calcium and magnesium ions. The laundering test was done according to the standard test method AATCC 124-1996 at a temperature of 46 C for 5 cycles of washing with AATCC standard detergent [28,29].

The antibacterial effect was determined by the standard method AATCC-100 with two bacteria (Escherichia Coli and staphylococcus aureus) with the agar plate method, and duration of bacteria growth was 24 hours. After 24 hours of bacteria growth, photos were taken of the agar plates, then colonies were counted and bacteria reduction was calculated from Equation (3):

Reduction (%) = (C − A/C) × 100
(3)

where C is the counted colonies on the cotton fabric; and A is the counted colonies of Ag loaded laser treated cotton.

For the determination of Ag ion content ICP-OES (Inductively coupled plasma optical emission spectroscopy) was used. The ICP-OES analysis of silver nanoparticle coated cotton fabric was carried out using a Varian Vista-Pro (Varian, Inc., Palo Alto, CA, USA) instrument. Each sample of cotton fabric weighing 0.05 grams was placed in a furnace (Nabertherm, Lilienthal, Germany) at a temperature of 600 °C for 1 hour, then nitric acid (10 ml) was added and distilled water to a volume of 25 ml. The Ag ion concentration was calculated from the calibration curve of standard solutions as was reported [30,31]. Scanning Electron microscopy (SEM) was performed to investigate the surface morphology of the untreated and laser treated cotton fabrics. The SEM microscope was a PHILIPS XL30 model and micrographs were taken at an acceleration voltage of 20 kV at magnifications of ×5,000, 10,000 and 15,000. EDAX analysis of SEM photos was performed to determine the amount of silver ions. Infrared spectra were collected using a Bruker-Equinox 55 system FTIR⁄ATR (Attenuated Total Reflectance) spectrometer. All data were recorded by means of a ZnSe Internal Reflective Element. Spectra were collected at a resolution of 4 cm^−1^ and 32 scans.

The carboxyl group content of cotton fibers was measured by the TAPPI standard test method. The untreated and laser treated cotton fabrics were dissolved in dilute hydrochloric acid, then washed and reacted with sodium bicarbonate , sodium chloride and then filtered. The filtrate was titrated with hydrochloric acid (0.01 M) in the presence of methyl red. The carboxyl group content was measured and reported in 100 grams of fabric in terms of meq (milli equivalents) [32].

## 4. Conclusions

Surface modification of cotton by a laser creates carboxylic acid functional groups. These groups are able to attract positively charged groups, so that they are absorbed by methylene blue cationic dye. The positive ions of metals such as silver are also able to attract the carboxylic acid groups. Therefore, silver nano particles are absorbed on the surface of laser treated cotton fabric and the increased uptake of silver nano particles on the surface increases the antibacterial properties so that the properties remain after repeated laundering.
